# Maternal Dyslipidemia and Its Association With Preterm Birth and Other Adverse Perinatal Outcomes

**DOI:** 10.7759/cureus.86599

**Published:** 2025-06-23

**Authors:** Mona Mishra, Neetu Singh, Ayush Lohiya, Manish Kulshrestha

**Affiliations:** 1 Department of Obstetrics and Gynecology, Dr. Ram Manohar Lohia Institute of Medical Sciences, Lucknow, IND; 2 Department of Public Health, Super Speciality Cancer Institute &amp; Hospital, Lucknow, IND; 3 Department of Biochemistry, Dr. Ram Manohar Lohia Institute of Medical Sciences, Lucknow, IND

**Keywords:** dyslipidemia, high-risk pregnancy, lipid, lipid disorders, lipid profiles, maternal and neonatal outcome, preterm labor, preterm labour

## Abstract

Background

The existing literature has reported a conflicting relationship between maternal dyslipidemia and preterm birth. No significant research has been performed to determine the association between maternal dyslipidemia and low birth weight (LBW), neonatal intensive care unit (NICU) stay, need for neonatal phototherapy, need for assisted ventilation, and stillbirths. This study aims to determine the association between maternal dyslipidemia and preterm births and other adverse perinatal outcomes.

Methodology

A total of 159 pregnant women were included in this prospective observational study. Serum lipid profile (triglycerides, total cholesterol, high-density lipoprotein, low-density lipoprotein, and very low-density lipoprotein) was tested once between 20 and 28 weeks of gestation. Mothers were followed throughout the antenatal period until delivery, and pregnancy outcomes such as preterm birth, LBW, NICU stay, need for neonatal phototherapy, need for assisted ventilation, and stillbirths were recorded in both dyslipidemic and eulipidemic women.

Results

In total, seven (8.8%) women with dyslipidemia had preterm birth, and five (6.3%) women with eulipidemia had preterm birth (p = 0.69). Moreover, 14 women with dyslipidemia had neonatal LBW, which was significantly higher compared to six women in the eulipidemia group (17.5% vs. 7.6%; p = 0.06). The number of women whose babies needed NICU stay was 10 (12.5%) and 1 (1.3%) in the dyslipidemia and eulipidemia groups, respectively (p = 0.005). The need for phototherapy was significantly higher in the dyslipidemia group compared to the eulipidemia group (5% vs. 0%; p = 0.045). In total, seven women in the dyslipidemia group needed neonatal assisted ventilation compared to one patient in the eulipidemia group (8.8% vs. 1.3%; p = 0.064).

Conclusions

Maternal dyslipidemia is not associated with increased preterm birth. However, maternal dyslipidemia is associated with a significant increase in other adverse neonatal outcomes such as LBW, increased NICU stay, and neonatal phototherapy.

## Introduction

According to the World Health Organization, 15 million babies are born preterm every year, accounting for 11% of pregnancies worldwide [1]. In 2010, 23.6% of global preterm births occurred in India, making India the leading contributor to the number of preterm births globally [2]. The prevalence of preterm birth is 13% in India, which is higher than that of its neighboring countries, such as Myanmar, Sri Lanka, China, and Nepal [3].

Preterm birth is a significant cause of death in children under the age of five years [1]. It is associated with various complications such as respiratory distress syndrome, hyperbilirubinemia, bronchopulmonary dysplasia, intraventricular hemorrhage, sepsis, and necrotizing enterocolitis. Long-term sequelae include coronary heart disease, type 2 diabetes, metabolic syndrome, and stroke in later life [4]. Growing evidence suggests that preterm birth is associated with death from infancy to adulthood [5].

The etiology of preterm birth is multifactorial. However, in almost half of all cases, no cause can be identified [1,6]. Various studies have investigated the role of maternal dyslipidemia in contributing to preterm birth [7-10]. However, the results have been inconclusive. Moreover, the magnitude of association and the effect of individual lipid components have been inconsistent across studies. The present study aims to investigate the association between maternal dyslipidemia and subsequent preterm birth. The association of maternal dyslipidemia with adverse perinatal outcomes such as low birth weight (LBW), neonatal intensive care unit (NICU) admission, neonatal phototherapy, and need for neonatal assisted ventilation was also determined.

## Materials and methods

Study design and site

A prospective, observational, analytical study was conducted in a tertiary care hospital in North India. Institutional ethical committee clearance was obtained before commencing the study (registration number: IEC No. 197/22). The study was conducted over one year (May 2023 to April 2024) in the antenatal outpatient clinic.

Sample size calculation

The sample size was calculated assuming a 15% prevalence of preterm births among pregnant females with normal lipid levels, a relative risk of 1.6, a precision of 0.5, an alpha error of 5%, and a power of 80% [6]. Assuming a 20% non-response rate, the sample size was calculated at 175 for the study.

Study population and eligibility criteria

Every consecutive pregnant woman between 20 and 28 weeks of gestation attending the antenatal outpatient clinic was assessed for eligibility. Women aged 18 to 40 years with a singleton pregnancy and willing to participate were included in the study. Women with multiple pregnancies, preeclampsia, gestational hypertension, chronic hypertension, type 2 diabetes mellitus, renal failure, gross congenital anomaly in the fetus, hepatic failure, obstructive liver disease, and those taking anabolic steroids or corticosteroids were excluded from the study.

Recruitment and enrollment

Women eligible for the study were recruited after obtaining written informed consent. The basic demographic information of the women was recorded, including age, education status, parity, socioeconomic status, dietary preferences, and body mass index (BMI). A detailed history and physical examination were conducted as per routine protocol on the day of recruitment. Blood samples for a lipid profile (triglycerides (TGs), total cholesterol, high-density lipoprotein (HDL), low-density lipoprotein (LDL), and very low-density lipoprotein (VLDL)) were collected at 20-28 weeks of gestation after obtaining written informed consent. Women were categorized into two groups based on their lipid profile. Women with a normal lipid profile were included in the first group, and women with dyslipidemia were included in the second group.

Definition of exposure

Dyslipidemia was defined as any one of the following: serum total cholesterol ≥200 mg/dL, serum TGs ≥150 mg/dL, serum LDL ≥130 mg/dL, serum VLDL >50 mg/dL, and serum HDL<40 mg/dL. These cut-offs are per the National Cholesterol Education Program( NCEP) Adult Treatment Panel III (ATP III) [11].

Lipid profile testing

A 2 mL venous sample was collected from the women after an overnight fast of ≥8 hours. The sample was centrifuged at 3,000 rpm for 10 minutes to yield serum. It was analyzed on Beckman Coulter AU480 using commercial kits from the same manufacturer via enzymatic spectrophotometric assays.

Study outcomes

The primary outcome was preterm births, defined as spontaneous birth at <37 weeks of gestation. The secondary outcomes in the study were neonatal admission to the NICU for more than 24 hours, LBW defined as neonatal birth weight less than 2.5 kg, need for assisted ventilation in the form of continuous positive airway pressure or ventilator support in the first seven days of life, and neonatal jaundice and need for phototherapy in the first seven days of life.

Follow-up of participants

Both groups of women received routine antenatal care as per the hospital protocol on subsequent follow-up visits. Complete obstetric history and examination were obtained at each antenatal visit, and routine obstetric investigations were done. All women were followed from the day of recruitment until the day of delivery. Pregnancy outcomes were recorded such as the route of delivery (vaginal versus lower-segment cesarean section), preterm birth, neonatal birth weight, neonatal NICU stay, need for phototherapy, and assisted ventilation.

Statistical analysis

Descriptive analysis was performed, and the results were presented as percentages with 95% confidence intervals (CIs). Quantitative variables were expressed as means with standard deviation. The proportion of preterm births among pregnant females with and without hyperlipidemia was calculated. The odds ratio (OR) of preterm birth among dyslipidemic women was calculated with a 95% CI. The proportions of preterm births among dyslipidemia and eulipidemia groups were compared using the chi-square test. Likewise, the means of the two groups were compared using an unpaired t-test. Associations were reported regarding unadjusted and adjusted ORs and 95% CIs. Pearson’s correlation was calculated to assess the association between two continuous variables. A p-value <0.05 was considered statistically significant. All analyses were performed using SPSS version 25 (IBM Corp., Armonk, NY, USA).

## Results

A total of 175 women were approached for the study. However, 14 participants were excluded because the follow-up data regarding the birth outcomes were not available. Two participants refused to participate in the study. Therefore, the data of 159 participants were included in the study. In total, 80 women were in the dyslipidemia group, and 79 were in the eulipidemia group. The mean (SD) age was 27.60 ± 4.10 years in the dyslipidemia group and 26.78 ± 3.64 years in the eulipidemia group (p = 0.188). The mean neonatal birth weight was 2.9 ± 0.48 kg and 3.0 ± 0.35 kg in dyslipidemia and eulipidemia groups, respectively (p = 0.33).

A comparison of the sociodemographic profiles of women in the dyslipidemia and eulipidemia groups across various factors, including religion, domicile, socioeconomic status, education, diet, and profession, revealed a significant difference (p = 0.044) in education level (Table 1). The proportion of individuals with higher education (70.0%) was higher in the dyslipidemia group than in the eulipidemia group (48.1%). Similarly, the proportion of individuals with a middle education was more prevalent in the eulipidemia group (39.2%) than in the dyslipidemia group (21.2%). Women in the dyslipidemia and the eulipidemia group were similar in terms of other factors such as religion, domiciliary status, socioeconomic status, and dietary preferences (Table 1).

**Table 1:** Comparison of sociodemographic and clinical profiles of patients in the dyslipidemia and eulipidemia groups. BMI: body mass index; NVD: normal vaginal delivery; LSCS: lower-segment cesarean section; CI: confidence interval

Variables		Groups		Odds ratio	95% CI	P-value
		Dyslipidemia, N = 80 (%)	Eulipidemia N = 79 (%)			
Socioeconomic	Lower	0 (0%)	1 (1.3%)	-	-	0.610
	Lower middle	44 (55.0%)	39 (49.4%)	-	-	
	Upper lower	29 (36.3%)	29 (36.7%)	-	-	
	Upper middle	7 (8.7%)	10 (12.6%)	-	-	
Education	Illiterate	1 (1.3%)	1 (1.2%)	-	-	0.044
	Lower	6 (7.5%)	9 (11.3%)	-	-	
	Middle	17 (21.2%)	31 (39.2%)	-	-	
	Higher	56 (70.0%)	38 (48.1%)	-	-	
Diet	Veg	57 (71.3%)	51 (64.6%)	1.361	0.697-2.655	0.366
	Non-veg	23 (28.7%)	28 (35.4%)			
BMI	Underweight (<18.5 kg/m^2^)	3 (3.8%)	3 (3.8%)	-	-	0.271
	Normal weight (>18.5–22.9 kg/m^2^)	30 (37.5%)	33 (41.8%)	-	-	
	Overweight (23–27.5 kg/m^2^)	34 (42.5%)	38 (48.1%)	-	-	
	Obesity (<27.5 kg/m^2^)	13 (16.3%)	5 (6.3%)	-	-	
Gestation of recruitment	Second trimester	69 (86.2%)	74 (93.7%)	0.424	0.140-1.282	0.120
	Third trimester	11 (13.8%)	5 (6.3%)			
Gestation of sampling	Second trimester	62 (77.5%)	73 (92.4%)	0.283	0.106-0.757	0.009
	Third trimester	18 (22.5%)	6 (7.6%)			
Mode of delivery	NVD	26 (32.5%)	40 (50.6%)	0.469	(0.247-0.893)	0.020
	LSCS	54 (67.5%)	39 (49.4%)			

The eulipidemia group had a significantly higher proportion of participants sampled in the second trimester (92.4%) compared to women with dyslipidemia (77.5%). In comparison, the dyslipidemia group had a larger proportion sampled in the third trimester (22.5%) than eulipidemia (7.6%) (Table 1). Women in the dyslipidemia group had a significantly higher cesarean rate than women with eulipidemia (67.5% vs. 49.4%; p = 0.02) (Table 1). No statistically significant difference was found for BMI, parity, indication for cesarean section, and other comorbidities between the groups.

The correlation coefficient was calculated to determine the association between lipid components and the period of gestation in which the levels were sampled. Serum TGs, cholesterol, and VLDL had a significant positive correlation with the period of gestation of sampling, whereas serum cholesterol showed a significant negative correlation (-0.15) with birth weight (p = 0.05) (Table 2).

**Table 2:** Pearson correlation between lipid components and BMI, birth weight, and gestation of sampling. BMI: body mass index; LDL: low-density lipoprotein; VLDL: very low-density lipoprotein; HDL: high-density lipoprotein

Variable	BMI		Birth weight		Gestation of sampling	
	Coefficient	P-value	Coefficient	P-value	Coefficient	P-value
Cholesterol	0.077	0.335	-0.153	0.054	0.229	0.004
Triglyceride	0.196	0.013	-0.010	0.897	0.229	0.004
LDL	0.113	0.158	-0.034	0.671	0.178	0.025
VLDL	0.176	0.027	-0.033	0.675	0.229	0.004
HDL	0.036	0.654	-0.089	0.263	0.001	0.985

The proportion of preterm births was higher in the dyslipidemia group (8.8%) compared to the eulipidemia group (6.3%). However, this was not statistically significant (p = 0.56). Preterm births were more common in women with TGs ≥150 mg/dL (8.3% vs. 6.7%; p = 0.691) and LDL ≥130 mg/dL (9.7% vs. 7%; p = 0.61) compared to women with TG and LDL levels less than these cut-offs. However, the results were not statistically significant (Table 3).

**Table 3:** Comparision of preterm births in the dyslipidemia and eulipidemia group. TG: triglyceride; LDL: low-density lipoprotein; VLDL: very low-density lipoprotein; HDL: high-density lipoprotein; CI: confidence interval

Exposure	Outcome		Odds ratio	95% CI	P-value
	Preterm, n (%)	Term, n (%)			
Dyslipidemia (n = 80)	7 (8.8%)	73 (91.2%)	1.419	(.431-4.676)	.563
Eulipidemia (n = 79)	5 (6.3%)	74 (93.7%)			
Total cholesterol ≥200 mg/dL (n = 71)	7 (9.9%)	64 (90.1%)	1.816	0.551-5.986	0.322
Total cholesterol <200 mg/dL (n = 88)	5 (5.7%)	83 (%)			
TG ≥150 mg/dL (n = 84)	7 (8.3%)	77 (91.7%)	1.273	0.386-4.194	0.691
TG <150 mg/dL (n = 75)	5 (6.7%)	70 (93.3%)			
LDL ≥130 mg/dL (n = 31)	3 (9.7%)	28 (90.3%)	1.417	0.360-5.575	0.617
LDL<130 mg/dL (n = 128)	9 (7.0%)	119 (93.0%)			
VLDL ≥50 mg/dL (n = 13)	1 (7.7%)	12 (92.3%)	1.023	0.121-8.61	0.984
VLDL <50 mg/dL (n = 146)	11 (7.5%)	135 (92.5%)			
HDL ≥40 mg/dL (n = 146)	12 (8.2%)	134 (91.8%)	1.097	1.043-1.154	0.282
HDL <40 mg/dL (n = 13)	0 (0.0%)	13 (100%)			

A higher number of women with dyslipidemia had neonatal LBW compared to women with eulipidemia. This finding was not statistically significant (17.5% vs. 7.6%; p = 0.06). Interestingly, the subgroup of women with TGs ≥150 mg/dL had significantly higher LBW neonates compared to those with TGs <150 mg/dL (17.9% vs. 6.7%; p = 0.034). No statistically significant difference was found for total cholesterol, LDL, VLDL, and HDL among the groups (Table 4).

**Table 4:** Comparison of birth weight in the dyslipidemia and eulipidemia group. TG: triglyceride; LDL: low-density lipoprotein; VLDL: very low-density lipoprotein; HDL: high-density lipoprotein; CI: confidence interval

Exposure	Outcome		Odds ratio	95% CI	P-value
	Birth weight				
	<2.5 kg, n (%)	≥2.5 kg, n (%)			
Dyslipidemia (n = 80)	14 (17.5%)	66 (82.5%)	2.581	0.937-7.105	0.060
Eulipidemia (n = 79)	6 (7.6%)	73 (92.4%)			
Total cholesterol ≥200 mg/dL (n = 71)	12 (16.9%)	59 (83.1%)	2.034	0.782-5.289	0.140
Total cholesterol <200 mg/dL (n = 88)	8 (9.1%)	80 (90.9%)			
TG ≥150 mg/dL (n = 84)	15 (17.9%)	69 (82.1%)	3.043	1.049-8.832	0.034
TG <150 mg/dL (n = 75)	5 (6.7%)	70 (93.3%)			
LDL ≥130 mg/dL (n = 31)	5 (16.1%)	26 (83.9%)	1.449	0.483-4.345	0.506
LDL<130 mg/dL (n = 128)	15 (11.7%)	113 (88.3%)			
VLDL ≥50 mg/dL (n = 13)	1 (7.7%)	12 (92.3%)	0.557	0.068-4.531	0.579
VLDL <50 mg/dL (n = 146)	19 (13.0%)	127 (87.0%)			
HDL ≥40 mg/dL (n = 146)	18 (12.3%)	128 (87.7%)	0.773	0.158-3.377	0.750
HDL <40 mg/dL (n = 13)	2 (15.4%)	11 (84.6%)			

The NICU stay of neonates was significantly higher in women with dyslipidemia than in women with eulipidemia (12.5% vs. 1.3%; p = 0.005). This was particularly significant in women with TG levels ≥150 mg/dL. The NICU stay was 10.7% in women with TG levels ≥150 mg/dL compared to 2.7% in women with TG levels <150 mg/dL (p = 0.046). No significant difference was seen in the NICU stay rate between women with normal and abnormal levels of total cholesterol, LDL, VLDL, and HDL (Table 5).

**Table 5:** Comparison of NICU stay in the dyslipidemia and eulipidemia group. NICU: neonatal intensive care unit; TG: triglyceride; LDL: low-density lipoprotein; VLDL: very low-density lipoprotein; HDL: high-density lipoprotein; CI: confidence interval

Exposure	Outcome		P-value	Odd ratio	95% CI
	Neonates needing NICU stay				
	Yes %	No %			
Dyslipidemia (n = 80)	10 (12.5%)	70 (87.5%)	0.005	0.09	0.011-0.790
Eulipidemia (n = 79)	1 (1.3%)	78 (98.7%)			
Total cholesterol ≥200 mg/dL (n = 71)	8 (11.3%)	63 (88.7%)	0.064	0.278	0.071-1.090
Total cholesterol <200 mg/dL (n = 88)	3 (3.4%)	85 (96.6%)			
TG ≥150 mg/dL (n = 84)	9 (10.7%)	75 (89.3%)	0.061	0.228	0.048-1.093
TG <150 mg/dL (n = 75)	2 (2.7%)	73 (97.3%)			
LDL ≥130 mg/dL (n = 31)	2 (6.5%)	29 (93.5%)	0.909	1.097	0.225-5.351
LDL<130 mg/dL (n = 128)	9 (7.0%)	119 (93.0%)			
VLDL ≥50 mg/dL (n = 13)	2 (15.4%)	11 (84.6%)	0.209	0.361	0.069-1.883
VLDL <50 mg/dL (n = 146)	9 (6.2%)	137 (93.8%)			
HDL ≥40 mg/dL (n = 146)	9 (6.2%)	137 (93.8%)	0.209	2.768	0.531-14.421
HDL <40 mg/dL (n = 13)	2 (15.4%)	11 (84.6%)			

A significantly higher number of newborns in the dyslipidemia group required assisted ventilation than in the eulipidemia group (8.8% vs. 1.3%; p = 0.064). No significant difference was seen in the need for assisted ventilation when individual lipid components were deranged.

The need for neonatal phototherapy in dyslipidemia and eulipidemia groups was 5% and 0%, respectively (p = 0.045). A subgroup of women with VLDL ≥50 mg/dL had 15.4% of their newborns needing phototherapy compared to 1.4% in women with VLDL <50 mg/dL (p = 0.002). No statistically significant difference was found for total cholesterol, triglycerides, LDL, and HDL.

Women with dyslipidemia had a similar stillbirth rate (1.3% vs. 1.3%; p = 0.27) compared to women with eulipidemia. Individual lipid components did not have a significant association with stillbirths.

We performed logistic regression of age, gestation at sampling, BMI, diet, still birth, NICU stay, assisted ventilation, LBW, cholesterol, triglyceride, LDL, VLDL, and HDL using the backward logistic regression method. It was found that NICU stay was significantly associated with preterm birth (p = 0.013). No other exposure had a significant association with preterm birth.

## Discussion

The principal findings of this study were that the occurrence of preterm births was not statistically different between the dyslipidemia and eulipidemia groups (8.8% vs. 6.3%; p = 0.69). Maternal dyslipidemia had a significant positive correlation with LBW neonates (17.5% vs. 7.6%; p = 0.06) and NICU stay (12.5% vs. 1.3%; p = 0.005). Maternal dyslipidemia was significantly associated with an increased need for phototherapy (5% vs. 0%; p = 0.045). There was no statistical difference in neonatal assisted ventilation between the groups (8.8% vs. 1.3%; p = 0.064) (Table 6).

**Table 6:** Comparison of preterm births and neonatal outcomes in women with dyslipidema and eulipidemia. NICU: neonatal intensive care unit; CI: confidence interval

Outcome	Dyslipidemia (n = 80)	Eulipidemia (n = 79)	Odds ratio	95% CI	P-value
Preterm birth	7 (8.8%)	5 (6.3%)	1.419	0.431-4.676	0.563
Low birth weight	14 (17.5%)	6 (7.6%)	2.581	0.937-7.105	0.06
NICU stay	10 (12.5%)	1 (1.3%)	0.090	0.011-0.719	0.005
Meconium-stained liquor	9 (11.3%)	4 (5.1%)	0.421	0.124-1.428	0.155
Neonatal phototherapy	4 (5%)	0 (0%)	0.950	0.903-999	0.044
Stillbirths	1 (1.3%)	1 (1.3%)	1.013	0.062-16.480	0.993
Assisted ventilation	7 (8.8%)	1 (1.3%)	7.479	0.898-62.279	0.064

A significantly higher proportion of dyslipidemic women were sampled in the third trimester compared to eulipidemic women, who had a higher proportion of women tested in the second trimester (Table 1). This can be explained by the fact that as the period of gestation increases, the lipid profile values get further deranged [12]. Hence, a higher proportion of women with deranged lipid values were in the third trimester of their pregnancy.

Mudd et al. reported that low total cholesterol, HDL-cholesterol, and LDL-cholesterol were associated with an increased risk of medically indicated preterm delivery. On the other hand, high total cholesterol, LDL-cholesterol, and TGs were associated with an increased risk of spontaneous preterm birth [12]. Contrary to Mudd et al., Edison et al. found low cholesterol levels (<10th centile) to be associated with preterm birth in low-risk white mothers [13]. Catov et al. concluded that high and low total cholesterol levels were significantly associated with preterm births (U-shaped relation). However, another prospective cohort study concluded that no U-shaped correlation exists between serum lipids and preterm birth [14].

Heida et al. found no correlation between unfavorable lipid profiles and spontaneous preterm birth [15]. On the contrary, Wang et al. found a significant association between raised total cholesterol, triglycerides, LDL, and HDL levels in the first trimester and preterm birth [16].

Thus, the results of the studies are conflicting, and the role of antenatal dyslipidemia in preterm birth is still controversial. A meta-analysis of 11 observational studies concluded that both low and high lipid levels are associated with preterm birth [9]. The study was associated with significant heterogeneity with I² values between 58.8% and 96.2% (p ≤ 0.05). In addition, out of 11 studies, only five provided the effect estimates of lower lipids. Due to fewer articles, the OR and relative risk values were pooled, resulting in the overestimation of effects between dyslipidemia and preterm birth. The results in various studies are different because of differences in gestation at which lipid testing was performed, as well as different lipid components being tested for the outcome. Further, some studies performed lipid sampling in the fasting state, while others considered non-fasting samples.

In the current literature, though a link is suggested between maternal dyslipidemia and preterm birth, there are still many unanswered questions regarding various factors, such as the optimal timing of lipid monitoring and the impact of different lipid components on preterm birth. There is significant heterogeneity in terms of the period of gestation when sampling was done. Some studies tested for the lipid profile as early as 15 weeks [8,17-20]. On the other hand, other observational studies studied lipid levels in the second and third trimesters [12,14,15,21-26].

The impact of various lipid components on preterm birth is still not fully understood. We found no significant association of individual lipid components, such as total cholesterol, triglyceride levels, LDL, HDL, and VLDL, with preterm births (Table 3). Some studies reported a positive correlation between raised HDL levels and preterm birth [24,26]. Other studies found that low cholesterol was significantly associated with preterm birth [13]. Some reported that raised cholesterol levels were associated with preterm birth [16,27]. These inconsistencies can be attributed to considerable heterogeneity among studies in terms of factors such as the gestation of sampling, fasting status, and the number of samples obtained. Moreover, different studies chose different lipid components to determine their association with preterm birth, which is also a possible explanation for such mixed results across the literature.

No study has yet found an association between abnormal lipid levels and other neonatal outcomes such as LBW, NICU stay, and the need for assisted ventilation. It is imperative to explore these associations. In our study, though raised lipid levels were not significantly associated with preterm birth, they were associated with a significant increase in other adverse neonatal outcomes such as LBW, NICU stay, need for phototherapy, and assisted ventilation (Table 6).

We found a significant association between raised lipid levels and the occurrence of LBW neonates. These findings conflicted with Okala et al., which suggested that raised total cholesterol from mid-pregnancy was associated with increased birth weight. High levels of HDL-cholesterol at 30 weeks were associated with fewer small for gestational age (SMA) babies, and low HDL-cholesterol was associated with a reduction in LBW babies [28]. In a study conducted by Kim et al., dyslipidemia was significantly associated with SGA babies [29]. It is explained by the hypothesis that raised lipid levels lead to atherosclerotic lipid accumulation in the placental bed. It also initiates an inflammatory cascade, thus decelerating nutrient transfer and lipids to the growing fetus and reducing weight gain of the fetus [30]. In our study, we found that the birth weight was lower in women with dyslipidemia, especially concerning TG levels. However, we did not calculate the number of SGA babies contributing to this, which is a limitation of our study.

Our study had various strengths. This study is the first of its kind to assess various adverse perinatal outcomes, such as preterm birth, NICU stay, LBW, stillbirths, and neonatal assisted ventilation, associated with elevated lipid levels. To our knowledge, no study has assessed the association between maternal dyslipidemia and these outcomes. We used cut-offs per the NCEP ATP III to define dyslipidemia, as there are no pregnancy-specific criteria to define dyslipidemia in pregnancy. Instead of using percentiles like previous studies [12,17], using these cut-offs prevented the inclusion of such women in the dyslipidemia group who did not have elevated lipid levels. We used fasting levels to avoid erroneously elevated lipid levels due to recent meals.

However, our study was not without limitations. The main limitation of the study is that, similar to many previous studies [12-14], we did a single test for lipid profile. Thus, we could not describe the trajectory of lipid levels throughout pregnancy. Single sampling may give misleading data, but we could not perform serial measurements due to social and financial constraints. Though it is known that lipid levels increase as gestation advances, we could not determine if the lipid levels were elevated at the time near delivery. Second, the sample size was small, and further robust, well-designed studies with a large sample size are required to investigate the effect of maternal dyslipidemia on preterm birth. Third, we took the same cut-offs to define dyslipidemia in both the second and third trimesters. This could affect the validity of our results as lipid levels increase with gestation. However, to counteract this, we performed multivariate analysis, which showed that the gestation of sampling had no significant association with preterm birth. This was despite the fact that lipid levels significantly increased with the period of gestation of sampling in our study, as determined by the Pearson correlation coefficient.

## Conclusions

Maternal dyslipidemia is not associated with increased preterm birth. However, it is associated with a significant increase in other adverse neonatal outcomes such as LBW, increased NICU stay, and neonatal phototherapy. Due to the small sample size, further large-scale studies are needed to confirm these findings.
